# Integrating complementary and alternative medicine into mainstream healthcare services: the perspectives of health service managers

**DOI:** 10.1186/1472-6882-14-167

**Published:** 2014-05-22

**Authors:** Judy Singer, Jon Adams

**Affiliations:** 1 University Centre for Rural Health, University of Sydney, PO Box 3074, Lismore NSW 2480, Australia; 2 University of Technology Sydney, Level 7, Building 10, 235-253 Jones St, Ultimo, 2007 NSW, Australia

**Keywords:** Integrative healthcare, Integrative medicine, Complementary and alternative medicine (CAM), Health services research, Chronic disease management, Psychological trauma management, Drug and alcohol rehabilitation

## Abstract

**Background:**

Complementary and alternative medicine (CAM) is increasingly included within mainstream integrative healthcare (IHC) services. Health service managers are key stakeholders central to ensuring effective integrative health care services. Yet, little research has specifically investigated the role or perspective of health service managers with regards to integrative health care services under their management. In response, this paper reports findings from an exploratory study focusing exclusively on the perspectives of health service managers of integrative health care services in Australia regarding the role of CAM within their service and the health service managers rational for incorporating CAM into clinical care.

**Methods:**

Health service managers from seven services were recruited using purposive and snowball sampling. Semi-structured interviews were conducted with the health service managers. The services addressed trauma and chronic conditions and comprised: five community-based programs including drug and alcohol rehabilitation, refugee mental health and women’s health; and two hospital-based specialist services. The CAM practices included in the services investigated included acupuncture, naturopathy, Western herbal medicine and massage.

**Results:**

Findings reveal that the health service managers in this study understand CAM to enhance the holistic capacity of their service by: filling therapeutic gaps in existing healthcare practices; by treating the whole person; and by increasing healthcare choices. Health service managers also identified CAM as addressing therapeutic gaps through the provision of a mind-body approach in psychological trauma and in chronic disease management treatment. Health service managers describe the addition of CAM in their service as enabling patients who would otherwise not be able to afford CAM to gain access to these treatments thereby increasing healthcare choices. Some health service managers expressly align the notion of treating the whole person within a health promotion model and focus on the relevance of diet and lifestyle factors as central to a CAM approach.

**Conclusions:**

From the perspectives of the health service managers, these findings contribute to our understanding around the rationale to include CAM within mainstream health services that deal with psychological trauma and chronic disease. The broader implications of this study can help assist in the development of health service policy on CAM integration in mainstream healthcare services.

## Background

Complementary and alternative medicine (CAM) is increasingly included within mainstream integrative healthcare (IHC) services for example [1-4]. To date, much of the focus in integrative health care research has aimed to: ‘identify factors related to successful integration of CAM and conventional medicine’ [5: 33]; to develop theoretical frameworks to explain the various models of integrative health care [5-9]; and to establish guidelines for effective integrative health care practice [10-12]. Significantly, the vast majority of this literature has focused on the perspectives of patients and practitioners, overlooking the role of health service managers in the practice of effective integrative health care [13].

Within the integrative health care literature, scholars increasingly report CAM to be popular and/or efficacious for the treatment and management of chronic health conditions [1,14-18]; and especially for the treatment of pain and stress related conditions [19-23]. Burke [24]: 932] argues that CAM may provide ‘potentially more effective multidisciplinary approaches to treating complex, chronic health problems such as HIV, chronic pain, and addiction’. Grace and Higgs’ [14]: 954] research in primary healthcare settings reports that patients and practitioners alike understood that an integrative approach ‘filled gaps in the treatment effectiveness’ for people experiencing complex, chronic conditions. Similarly, research investigating CAM within integrative health care services in hospital settings found that CAM addressed therapeutic gaps in chronic disease management [2,20]. Indeed, it is well established that ‘individuals who have a chronic condition, particularly a mental health condition, are more likely to use CAM’ [25]: 371].

Significantly, the vast majority of the integrative health care research exploring the role of CAM in the treatment of chronic conditions has investigated the interface between biomedicine and CAM [2,10,19,26-30]. There has been minimal research investigating the practice of CAM alongside other disciplines such as psychology.

In this paper we broaden the scope of integrative health care from the sole collaboration between biomedicine and CAM [30] to include the therapeutic alliance of CAM with psychological therapies. We note that within the wider health domain a small number of qualitative studies exploring the role of CAM within community mental health services [4,31] and drug and alcohol programs [32] are emerging. Furthermore, there appears to be a relatively new development within the CAM and integrative health care literature with some scholars drawing on health promotion theory and practice to develop new frameworks to contextualise the potential role of CAM in public healthcare contexts, especially for the management of chronic diseases [17,33-36].

Despite health service managers’ being key stakeholders central to ensuring effective integrative health care, little research to date has specifically investigated the perspectives of health service managers with regards to integrative health care services under their management [13]. In response, this paper reports findings from an exploratory study that focuses exclusively on the perspectives of health service managers of integrative health care services in Australia regarding the role of CAM within their service and the health service managers rational for incorporating CAM into clinical care.

## Methods

The methods employed in this study were first reported in a previous paper [13]. The study focuses on the perspectives of health service managers who are responsible for the development, functioning and outcomes of their integrative health care services. These services are located across three Australian States and comprise five community health services and two hospital-based specialist services. In all services CAM was practiced alongside other disciplines (not necessarily mainstream medicine). In some services, clients could choose to access only CAM treatments, but it was not generally considered a ‘stand alone’ therapy.

### Study Sites: Community health service (CHS)

The community health services in this study dealt predominantly with the effects of trauma, including drug and alcohol misuse, sexual assault, domestic violence, and refugee trauma. Many clients attending these services were from low socio-economic backgrounds, often including culturally and linguistically diverse groups and Indigenous populations. What is noteworthy about these services is that they provide people access to CAM who would otherwise not be able to afford such treatments.

Notably, the health service managers in the community health services were not trained in CAM; their professional backgrounds included psychology and social work. They had been managing the integrative health care program in their service for between three and 14 years. Significantly, three of the community health services had included a CAM program for over 20 years.

The CAM practitioners comprised: naturopaths, traditional Chinese medicine practitioners, yoga and shiatsu practitioners and massage therapists. CAM was co-located with the other disciplines in the service. Tables 1 and 2 provide further details including referral processes, funding arrangements and longevity of the CAM programs.

**Table 1:** Community healthcare services: CAM programs

**Community health services**	**CHS 1**	CHS 2a and 2b**	**CHS 3**	**CHS 4**	**CHS 5**
Type of service	Drug and alcohol rehabilitation	Women’s health	Refugee mental health service	Drug and alcohol rehabilitation	Women’s health
Funding of CAM program/practitioners	Government funded; CAM salaried positions	Short term funding; Sessional positions	Government funded; CAM salaries allocated core funds	Government funded; CAM salaried positions	Government funded; CAM salaried positions
Referrals	In-house: nurse & counsellors; External: GPs & other services; Self-referral	In-house only: counsellor; No self-referral	In-house only from counsellors No self-referral	In-house: GPs, counsellors; External: GPs & other services; Self –referral	In-house: GPs, counsellors & nurse; External: GP & other services; Self-referral
Cost	Current clients free; ‘Gold coin’ donation (one/two dollars) ex-clients & low income	Free	Free	Free massage and acupuncture; low cost Chinese medicines	Free consultations low cost medications

**CHS 2a and 2b denotes the two workers that job-share managing the CAM program.

**Table 2:** Overview of hospital CAM programs

**Hospital**	**H1**	**H2**
Type of service	Adjunct to medical treatment; specific chronic disease	Adjunct to medical cancer treatment
Funding of CAM program/Practitioners	Grants and industry support	Charity status; reliant on donations
Referrals: in-house/external	Self-referred; In-house: nurses, physio, doctors	Self-referred; In-house: nurses, physiotherapists, doctors
Longevity of CAM program	Three years	Nine years
HSM: duration in position	Three years	Nine years
HSM: Professional background	Dual-trained: CAM & allied medical	Psychology & allied medical

### Study sites: Hospitals

Two hospitals with a CAM program were investigated: 1. CAM programs dealing with cancer care; 2. CAM programs dealing with an unspecified physical condition (to preserve anonymity, we refer to the second program as dealing with a chronic condition). Patients attending these programs were from a broad range of socio-economic and ethnic backgrounds.

Notably, the hospital integrative health care programs included only body-based CAM which were provided free of charge. The majority of the CAM workforce was either student practitioners or volunteers who were supervised by CAM professionals. In both hospitals CAM was practiced on the ward and in one hospital CAM was also available in a stand-alone clinic within the hospital (Table 2).

### Recruitment

The first author used existing professional networks and an internet search to identify public healthcare services in Australia that had a CAM program as part of service delivery. In order to reflect a cross-sectional scope of services, the sampling frame included diversity of patient populations and service type (community-based services and hospital); as well as geographic range through inclusion of services in three Australian states.

The health service managers responsible for the CAM programs in their service were recruited using criterion-based purposive and snowball sampling. All participants were senior in their field and had a minimum of 12 months clinical management experience. Initial contact was made via email and potential participants were informed about the study and invited to participate in a one hour interview. If interested, they were sent a participant information statement and consent form and an interview time was organised. The first few participants were asked if they could recommend other potential participants.

### Methodology

To investigate participants’ perspectives about the CAM programs in their service we conducted semi-structured interviews. This methodology enabled participants to reflect on their experiences and explore a range of topics. An initial review of the integrative health care literature was conducted in order to scope the field and to inform the interview guide. This included: the practice of CAM within mainstream health care settings; the rationale for including CAM; the referral processes between health professionals; and an exploration of notions of integrative health care.

### Data collection

Interviews were conducted in 2011 with eight service managers who chose to be interviewed in their workplace. In one of the services two counsellors job-shared the management of the CAM program and both participated in a joint interview. The health service managers’ dual role enabled these participants to speak from the position of manager and referring counsellor. This added significant depth to the interview as their understandings about the interface of CAM and counselling came from their clinical experience with the CAM practitioners. All participants were female and no potential participants declined to be interviewed. With consent the interviews were digitally recorded and then transcribed to computer file. To de-identify the data, during the transcribing process the participants were allocated pseudonyms. The study was granted ethical approval from the University of Sydney, Human Research Ethics Committee.

### Data analysis

Interviews were conducted, transcribed verbatim and carefully read and reread with the aim to identify broad concepts and categories. Based on the principles set out by Gifford [37] and Patton [38], a coding framework was developed and the transcripts were coded for dominant categories and themes. In order to systematically code the data a coding template was developed and each transcript was reorganised under relevant themes. This is a slow and intensive process that provides a methodical and structured approach to data coding. Journal notes were kept throughout the coding process, in order to document emerging themes, ideas and reflections.

Data were thematically analysed using Gifford’s [37]: 544] three step approach which includes: ‘description, classification and connection’. Description allows the data to be classified into meaningful categories in order to develop a conceptual framework with the aim to make connections between and within categories and to decipher variations [37]: 544].

## Results

**Figure 1:**
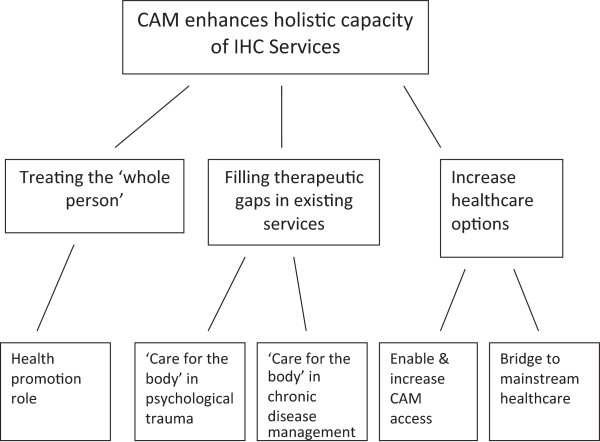
Health services managers’ rationale for including CAM in service delivery.

In this section we describe the main themes that emerged from the thematic analysis of participants’ perceptions about the role of CAM and their rationale for including CAM into clinical care.Figure 1 summarises the various ways in which the service managers explained how CAM improved holistic capacity.

The overarching theme to emerge from the analysis was a perception of the inclusion of CAM as enhancing the holistic capacity of health care services.

The health service managers in both community health services and hospitals stated that the inclusion of CAM enhanced the capacity of their organisation to deliver a holistic service.

‘We talked about bringing in some [CAM] therapies because there was interest in seeing [our service] become more holistic in terms of attending to different aspects of a person’ (CHS2a)

Health service managers perceived CAM to improve holistic capacity in three specific ways: through treating the ‘whole person’; by filling therapeutic gaps in existing service delivery; and via increasing healthcare options for patients.

### Treating the whole person

Connected with conceptions of holism, a core tenet of CAM as presented by some commentators is the understanding that CAM focuses on ‘treating the whole person’ [39]. The health service managers used the concept of ‘treating the whole person’ to explain their rationale for including CAM as part of the healthcare approach in their service, and to distinguish the role of CAM from that of other health practices in their service.

‘CAM practitioners do a lot more, they are in situations where they might find out a lot more about people’s stories and about their mood … rather than simply talking to them about diet’ (CHS1)

In one community health service the health service manager described the service’s approach to health care as underpinned by *‘the understanding that all of life impacts on health’ (CHS5).* The health service manager explained that the scope of health care practitioners, which included counsellors, nurse practitioners, doctors and CAM therapists, enabled *‘different ways of creating environments for health and well-being’ (CHS5).* In this health service manager’s service CAM was understood as a critical component of the holistic service delivery.

‘Health is intrinsically entwined with self and CAM [in this service] has always been seen as very important and very valid’ (CHS5)

As indicated in the quote below, a holistic model was seen to be well established within this particular organisation as CAM had been included since the organisation’s inception over 30 years ago.

‘[This service] is not a medical centre with CAM therapists; it is a health centre where all of the services are equal in importance … [we have] many disciplines under the one roof sharing and cross referring and [engaged in] coordinated care’ (CHS5)

Commonly, health service managers discussed the importance of diet and nutrition as being a critical component of a whole person/holistic approach. Participants expressed the view that CAM practitioners were better placed to address nutritional issues from a holistic perspective than other practitioners such as dieticians. As one health service manager explained; *‘CAM practitioners have added knowledge’ (CHS1).* In two instances (hospital and community health service), health service managers purposely employed CAM practitioners instead of dieticians as they perceived CAM practitioners to provide a holistic approach to nutrition.

‘We have purposefully employed CAM practitioners because they are trained to provide nutritional advice, or to prescribe a diet; and when I say “diet”, I don’t mean necessarily a sliming diet, but a proper, whole food, healthy nutritional approach’ (CHS5)

The health service managers understood that the CAM practitioners’ training enabled them to focus upon balancing diet and emphasising fresh, whole-foods, rather than disease-focused prescribing:

‘CAM training isn’t so much about dealing with frank deficiencies, but more so dealing with optimal health and well-being … what we are trying to do is to put in place a program of preventative health, so the goals are different to that of a dietician who is dealing with deficiencies and disease states’ (H1)

Another aspect of ‘treating the whole person’ identified by health service managers was the importance of the CAM case history. In one of the trauma services, the health service manager explained that CAM practitioners took an extensive case history from each client, including a detailed exploration of lifestyle factors and dietary habits. Written consent provided by the client enabled the counsellors and CAM practitioners to share important details regarding the client’s wellbeing.

One health service manager, who is also a counsellor in the service, explains that the CAM practitioner was able to identify significant issues about the client’s health behaviours which had gone undetected during the psychological assessment.

‘I had no idea that [my client] was drinking 20 cups of coffee a day; no wonder she was hyper-aroused … another client was living on cuppa soups and I had no idea, [diet] wasn’t something that got addressed in therapy’ (CHS2b)

By bringing the body into focus through CAM approaches to dietary habits, health service managers explained that clients received a ‘more holistic’ approach that they perceived made a difference to their well-being:

‘Feedback from the clients [showed] that some of their lifestyle choices and habits were shifting significantly and they were more mindful about making different choices around smoking, drinking and eating’ (CHS2b)

### Treating the whole person: CAM and health promotion

As described, the health service managers were knowledgeable about and drew upon a CAM holistic orientation in the interviews. Some health service managers framed this approach by describing CAM and its role in their service provision as underpinned by a health promotion model:

‘CAM practitioners have clinical expertise, they have the passionate interest in health promotion … within the clinical role and health promotion role they are exceptionally well trained … they know how to talk to a patient and to make them feel at ease. They can chat to them about diet and lifestyle and health promotion and help them to open up’ (H1)

This hospital health service manager explained that the rationale for framing the CAM practitioner’s role as congruent with ‘health promotion’ was a strategy to facilitate acceptance of CAM within a high-tech biomedical domain:

I think that there is still a sense among people that practicing [CAM] you might be taking people off their medication, or giving them conflicting advice; whereas health promotion is not controversial, [medical staff] see it as strengthening what they are doing, rather than conflicting’ (H1)

Moreover, a CAM approach to health promotion as described by the health service manager not only consisted of a crucial focus upon the interconnecting factors of diet, exercise and lifestyle but also required communication skills, including the ability to listen to patients.

‘CAM training means that they fully appreciate the role of diet and lifestyle within this context … they also understand how to communicate with patients’ (H1)

### CAM fills therapeutic gaps in the treatment of psychological trauma and chronic disease management

The second overarching theme to emerge from health service managers perspectives about the CAM role in enhancing holistic capacity in their service was filling ‘therapeutic gaps’ in service delivery. Health service managers perceived CAM to fill therapeutic gaps in services dealing with psychological trauma and chronic disease management by providing ‘care for the body’ (via body-based therapies; Traditional Chinese Medicine; Western herbal medicine and naturopathy). The health service managers explained that the CAM programs had been included into clinical care to attend to somatic and physical symptoms through a mind-body approach rather than a conventional medical one. For instance, CAM was included in hospital settings for patients undergoing cancer treatments and for some post-operative patients; in community health services providing drug withdrawal programs; and in conjunction with trauma-based counselling.

‘We take a holistic approach to contributing to recovery, because we know that with trauma the body is bruised as well as the mind, so it is appropriate to respond to the body as well as the mind, and just responding to the mind may not be enough’ (CHS3)

### CAM fills therapeutic gaps: ‘Care for the body’ in psychological trauma interventions

A recognition of the importance of providing appropriate mind-body approaches as part of the treatment protocols for addressing trauma was given by health service managers as the main clinical rationale for the inclusion of CAM in these services.

‘[The counsellors] have been saying “the body is harmed [by trauma], why aren’t we healing using the body” … CAM fills that gap and counsellors are seeing the value and benefit of it’ (CHS2a)

‘[Collaborative practice between counselling and CAM] *actively affirms the importance of the psychological and the physical, the mind and the body; it reminds counsellors that CAM can play a very important role in the healing and recovery for our client population (CHS5)*

‘Massage is about appropriate touch, about healing in a way that is professional and appropriate and yet also intimate. There is a level of trust from the client’s perspective, and so long as that is kept safe, it can be a very validating and positive experience for the client’ (CHS4)

In the following set of quotes the health service managers explain how CAM, when practiced in parallel with psychotherapy, has the potential to broaden the therapeutic outcomes of psychotherapy in the treatment of trauma.

Rationale for including the CAM program:

It’s a false divide between the body and the mind which has been the historical way that trauma has been seen. We are trying to bring the two fields together and have the value of both recognised …’ (CHS2a)

The health service managers perceived CAM therapies as providing the appropriate therapeutic strategies to literally bring the body into the therapeutic encounter, and thereby offset the limitations of a solely verbal approach. They understood CAM to attend to the body in ways that were outside the scope of practice of counsellors:

‘Verbal therapies are limited and we need something alongside the verbal … I felt that I was only going so far with verbal therapy and the client’s trauma had impacted in ways where her relationship with her body had been quite distorted … I felt that [after CAM the client] was more present in her body and more “up” in terms of body posture…[CAM enables clients to] experience touch in a bounded and safe way, and is seen as an opportunity for changing people’s experience [of touch] (CHS2b)

The health service managers, who were also counsellors in the service, described CAM to have therapeutic benefits beyond providing relaxation for clients:

Rather than CAM being just about relaxation, it is about affect regulation … it can offer relaxation, but it can also activate and engage the body in a different form of healing … with one of my clients [CAM] helped to reduce self harm behaviour … as we increased the CAM, her cutting behaviour was eliminated’ (CHS2b)

Moreover, the health service manager describes the range of therapeutic effects of CAM on clients:

When we [started the CAM] program we never would have identified specifically that [clients] would have better boundaries or become more assertive; things that you’d expect to happen from role playing in verbal therapies, but we wouldn’t necessarily expect to see that from the body based therapies, and it has come through quite consistently and strongly [in the feedback] … All the counsellors have the sense that clients are being able to regulate their emotions more … there are differences with clients around boundaries … clients are making big changes with diet and exercise and self-care … and it was surprising that a lot of the counsellors were noticing the same things (CHS2b)

In this case example, the health service managers’ depth of understanding about the role of CAM and the respectful collaborative integrative health care practice were fundamental to the efficacy of this integrative health care approach. Notably, the therapeutic value of CAM was understood to be more than the provision of relaxation or stress management techniques. Notwithstanding the therapeutic value of reducing stress, the health service managers’ explained how CAM significantly extended the scope of psychological treatment.

In addition, the health service managers suggest that CAM also fills cultural gaps in services dealing with psychological trauma. Health service managers described how CAM therapies often provide a culturally appropriate approach to care that was familiar to culturally diverse clients, and in some cases more relevant than ‘talk-based’ therapies, especially for dealing with the somatic presentations for these clients.

‘One woman in the yoga group was Vietnamese and she really wanted to do the yoga as she saw it as just as beneficial as the verbal therapies, and her therapist thought it would be good as the verbal was only going so far’ (CHS2b)

‘Clients from particular ethnic groups respond very well to CAM and don’t respond so well to counselling. CAM is a very important intervention in their recovery and healing’ (CHS3)

### CAM fills therapeutic gaps: ‘care for the body’ in chronic disease management

In addition to filling therapeutic gaps in psychological trauma interventions, some health service managers emphasised the ways in which CAM also enhanced well being by providing ‘care for the body’ in chronic disease management. Health service managers endorsed the use of CAM for their capacity to provide nourishment, relaxation and as an opportunity to receive therapeutic care.

‘All of the things that we offer and the way in which the [CAM practitioners] are trained; all of it creates a relaxation response’ (H2)

‘It’s the care of another person that’s important, as well as the relief [that patients get] from massage’ (CHS3)

Where patients were undergoing difficult medical treatments health service managers explained that CAM was seen to reduce the distressing physical effects of these treatments and to provide an opportunity to receive care and enable relaxation.

‘Patients who have been diagnosed [with cancer], no matter what stage of their diagnosis or treatment plan, are usually highly anxious and distressed and [CAM therapies] reduce that’ (H2)

In the hospital contexts, the health service managers described how the CAM practitioners had taken on a ‘caring role’ within the medical domain. It was noted by the health service manager’s that CAM practitioners took the time to talk to patients, listen to their concerns, inquire about their wellbeing, and provide therapeutic touch in the form of massage and other body-based therapies.

In both hospital settings, the health service managers stated that an identifiable gap in hospital-based care is the lack of time staff experience for developing therapeutic relationships with patients. The health service managers explained that an added benefit of the CAM role was that it could respond to this need, and as the health service manager explained in the quote below, nursing and physiotherapy staff were appreciative of the addition of CAM practitioners.

‘The therapeutic relationship is a missing niche … nurses don’t have time to hold a person’s hand and talk to them … the physios are so busy but they understand the role of massage therapy and that’s why we get so many referrals [from them]’ (H1)

### CAM increases healthcare options

#### Improving access to CAM

Health service managers explained that providing either a free or low cost CAM program alongside the free or low cost mainstream treatments within their service created the opportunity for clients to have access to a broader scope of healthcare options.

‘Being able to offer as much as possible for the client; we want to make sure that everyone has [CAM] available to them, we can’t miss that opportunity’ (CHS1)

‘Clients like CAM and it’s about being able to offer something outside [mainstream] treatment services. If we can increase the scope of what we are able to offer clients, then it might just be enough to make that much more of a difference’ (CHS4)

In the two hospital settings, CAM therapies were offered in conjunction with the patient’s medical treatment. . The health service manager’s view was that because the CAM treatments were readily available, free, located in the same site as the patients’ medical treatment, and endorsed by (most) hospital staff, this ensured greater accessibility. According to the health service managers the visibility and availability of CAM in these settings provided an opportunity for patients to access treatments that may otherwise not be available to them.

‘We have a separate stand-alone [CAM] centre within the hospital … we are on the main thorough-fare and we get a lot of people dropping in’ [H2]

In the community health services, health service managers described the access to CAM as linked to broader social justice commentary on the importance of providing equity in healthcare, which was also seen as a critical component of holistic care.

‘It’s about saying we want to provide the best service available and offer the widest range of possible interventions because you are worth it … people should be able to access CAM’ (CHS4)

This was particularly the case for services working with people from low socio-economic backgrounds, who would otherwise not be able to afford CAM treatments.

‘People shouldn’t have options denied to them because they can’t afford it. CAM in the community is usually very expensive; that’s the difference with us, we make it affordable’ (CHS5)

#### Bridge to mainstream healthcare

Particularly within the Drug and Alcohol rehabilitation services and those working with culturally diverse populations, health service managers explained that some clients experienced CAM as a more familiar and comfortable healthcare option than conventional medicine or counselling. As such, the health service managers in these services stated that CAM is often taken up by clients as a safe first point of contact with the service.

‘[For some clients] CAM does not come with the same label or tags or fears [as Western Medicine]’ (CHS4)

As one health service manager stated, beginning treatment with a CAM practitioner enabled clients to establish trust and rapport with the health service and often led to the client accepting a referral to the doctor or counsellor. As such, CAM acted as a ‘point of entry’ or bridge for vulnerable clients into mainstream healthcare.

‘The CAM role is not only about supporting the engagement and treatment, but it is a fantastic avenue to break down the barriers between clients and treatment services for those clients who are often resistant to treatment’ (CHS4)

In the services dealing with drug and alcohol rehabilitation CAM was seen by the health service managers to provide a point of entry for some clients who were resistant to conventional medicine or counselling. One health service manager described CAM to provide clients with ‘a foot in the door’ to the service, enabling them to build trust and feel comfortable attending the service.

‘My perception is that CAM is a great way [for clients] to get a foot in the door, to start the engagement as CAM is not intrusive’ (CHS4)

In situations where CAM is culturally more familiar than counselling, health service managers stated that having access to CAM within their service provided a more manageable segue to access other treatments.

‘There are a lot of clients who talk about the importance of herbal medicine because that has been part of what they have used in the past … there are the cross-cultural indicators that for many clients this is a familiar approach, and in the first instance, a more embraceable intervention than talking therapies’ (CHS3)

## Discussion

The health service managers interviewed in this study linked the inclusion of CAM in their services to notions of holism within integrative models of health care. They understood CAM to add ‘holistic capacity’ to their service by treating the whole person, by filling therapeutic gaps and by broadening the scope of healthcare options for patients. The health service managers universally reported that it was the specific inclusion of CAM therapies that increased the holistic value of their healthcare context.

Echoing our findings, a study exploring counsellors’ understandings and reasons for referral to CAM in a community-based mental health service argues that the creation of ‘integrative models of care that combine a range of different practitioners and modalities represents an attempt to create a more holistic model’ [40]: 12]. Similarly, in Keshet et al’s [2]: 586–7] research, investigating ‘whether treatment becomes more holistic when CM [complementary medicine] is integrated’, the researchers reported CM integration as providing a more ‘comprehensive’ approach to patient care.

Notably, in the present study, health service managers were able to articulate exactly how the inclusion of CAM enhanced the holistic value of their service and contributed therapeutic value. Although most health service managers had no formal CAM training, their knowledge proficiency of CAM was significant and this was evidenced in their understandings about the clinical effects of CAM in service delivery. As we have previously described, the health service managers sound understanding of the clinical applications of CAM within their service was seen as a key component for ensuring effective integrative practice [13].

The health service managers in this study understood CAM to fill specific therapeutic gaps within their service. The rationale for healthcare services to include a CAM program based on a recognised gap in service delivery is not well researched. However, a study by Kopansky-Giles and colleagues [20] notes that the recognition by management personnel of a specific therapeutic gap led to the inclusion of chiropractic in the hospital investigated in their study. Keshet et al’s [2] research reports that the hospital medical staff and senior administrators in their study understood CAM to ‘treat patients’ unmet needs, which they perceive mostly as psychological needs’ [2]:590].

Within the five community health services that dealt with various types of psychological trauma in our study, the health service managers understood CAM to fill gaps by providing mind-body approaches that were outside the scope of practice of medical and psychological treatments. Correlating these understandings is neuro-biological research in mainstream trauma interventions which acknowledges the need to incorporate the body in trauma work [41,42]. Health service managers understandings about the therapeutic role of CAM in trauma interventions is substantiated by Collinge and colleagues [31] who suggest that massage treatment for people experiencing depression and anxiety as a consequence of sexual abuse provides positive clinical outcomes. Corroborating findings from the present study, Collinge and colleagues [31] report that the inclusion of CAM improved the therapeutic progress in some difficult to treat cases. Although it might seem counter-intuitive to use massage with survivors of sexual assault, the authors note that massage treatment at the appropriate time and in conjunction with psychotherapy can provide substantial benefits [31].

While CAM is known to provide mind-body approaches in a range of health contexts, the inclusion in mental health services that deal specifically with trauma is uncommon. The findings in the present study suggest the collaborative practice between CAM and counselling is an effective therapeutic alliance, and this view is supported by earlier research [4,31,43].

A recent development in the CAM literature is the linkage of CAM with aspects of health promotion [17,36,44]. In the present study some health service managers conceptualise the role of CAM within their service from a health promotion model whereby the connection between diet and nutrition, relaxation and stress management are understood to be underpinned by a salutogenic orientation [45]. Discussed elsewhere, salutogenesis is a way of framing health and illness which is orientated toward investigating what creates health rather than focusing on the pathological causation of disease [45]: 8]. Data from our study suggests that one of the rationales given by the health service managers for including CAM into service delivery was the understanding that this approach was orientated to ‘prevention, health promotion, and patient empowerment’ [17]: 257].

Another rationale given by some of the health service managers for including CAM was to ensure better equity in healthcare choice for people from low socio-economic backgrounds. The importance of addressing issues of equity and social justice in healthcare provision is discussed by Nissen [46]: 63], who state that for many patients seeking CAM treatment ‘to relieve symptoms and promote well-being in chronic health conditions and life-threatening illnesses … a considerable barrier is that CAM is most often paid for out-of-pocket’. The integrative health care programs described in the present study provided free or low cost CAM and as the health service managers described, this ensured that CAM was financially accessible for people who would otherwise not afford these treatments.

### Study limitations

This study has several limitations: the small sample size is informative, but does not represent conclusive data about CAM integration; recruiting from existing professional networks incurs some level of sampling bias; and patient and practitioner perspectives linked to these services have not been examined in this study.

## Conclusion

The diversity of health care settings with a CAM program is evidence of the growing appreciation of CAM relevance and effectiveness in a broad range of healthcare contexts. The findings reported here provide insight into how health service managers of health services that include a CAM program explain the therapeutic effects or value of CAM within their service, how they conceptualise notions of holism and, in turn, how they perceive CAM to increase the holistic value of their service. Our findings contribute to understandings about the role of CAM in services that deal with chronic disease and builds on previous research by examining the role of CAM in various services that deal with psychological trauma. The broader implications of this study may assist in the development of health service policy on CAM integration in mainstream healthcare services.

## Abbreviations

IHC: Integrative healthcare; CAM: Complementary and alternative medicine; CHS: Community health service; H: Hospital.

## Competing interests

The authors declare they have no competing interests.

## Authors' contributions

JS developed the study design, collected the data, carried out the data analysis and drafted the original manuscript. JA contributed to the data analysis and interpretation of the findings, and revised and approved the final manuscript. Both authors read and approved the final manuscript.

## Pre-publication history

The pre-publication history for this paper can be accessed here:

http://www.biomedcentral.com/1472-6882/14/167/prepub
